# Supplementary material for: Resolving the full spectrum of human genome variation using Linked-Reads
Source: Genome Res. 2019 Apr;29(4):635–45. doi: 10.1101/gr.234443.118 (PMC6442396; doi:10.1101/gr.234443.118)
Supplement: Supplemental Material [file supp_gr.234443.118_Supplemental_longranger-2.2.zip › longranger-2.2.tar.bz2!/lr2.2-public/lib/stan/cmdstan/stan/doc/api/html/classstan_1_1gm_1_1argument.html]

Stan: stan::gm::argument Class Reference


|  |  |
| --- | --- |
|  | Stan  2.5.0  probability, sampling & optimization |


- Main Page
- Related Pages
- Namespaces
- Classes
- Files

- Class List
- Class Index
- Class Hierarchy
- Class Members

All Classes Namespaces Files Functions Variables Typedefs Enumerations Enumerator Friends Macros Pages

- stan
- gm
- argument

Public Member Functions |
Static Public Member Functions |
Protected Attributes |
List of all members

stan::gm::argument Class Referenceabstract

`#include <argument.hpp>`

Inheritance diagram for stan::gm::argument:

|  |  |
| --- | --- |
| Public Member Functions | |
|  | argument () |
|  | |
|  | argument (const std::string name) |
|  | |
| virtual | ~argument () |
|  | |
| std::string | name () const |
|  | |
| std::string | description () const |
|  | |
| virtual void | print (std::ostream \*s, const int depth, const std::string prefix)=0 |
|  | |
| virtual void | print\_help (std::ostream \*s, const int depth, const bool recurse)=0 |
|  | |
| virtual bool | parse\_args (std::vector< std::string > &args, std::ostream \*out, std::ostream \*err, bool &help\_flag) |
|  | |
| virtual void | probe\_args (argument \*base\_arg, std::stringstream &s) |
|  | |
| virtual void | find\_arg (std::string name, std::string prefix, std::vector< std::string > &valid\_paths) |
|  | |
| virtual argument \* | arg (const std::string name) |
|  | |
| int | compute\_indent (const int depth) |
|  | |

|  |  |
| --- | --- |
| Static Public Member Functions | |
| static void | split\_arg (const std::string &arg, std::string &name, std::string &value) |
|  | |

|  |  |
| --- | --- |
| Protected Attributes | |
| std::string | \_name |
|  | |
| std::string | \_description |
|  | |
| int | indent\_width |
|  | |
| int | help\_width |
|  | |

## Detailed Description

Definition at line 12 of file argument.hpp.

## Constructor & Destructor Documentation

|  |  |  |  |  |
| --- | --- | --- | --- | --- |
| stan::gm::argument::argument | ( |  | ) |  |

inline

Definition at line 15 of file argument.hpp.

|  |  |  |  |  |  |
| --- | --- | --- | --- | --- | --- |
| stan::gm::argument::argument | ( | const std::string | *name* | ) |  |

inline

Definition at line 19 of file argument.hpp.

|  |  |  |  |  |
| --- | --- | --- | --- | --- |
| virtual stan::gm::argument::~argument | ( |  | ) |  |

inlinevirtual

Definition at line 24 of file argument.hpp.

## Member Function Documentation

|  |  |  |  |  |  |
| --- | --- | --- | --- | --- | --- |
| virtual argument\* stan::gm::argument::arg | ( | const std::string | *name* | ) |  |

inlinevirtual

Reimplemented in stan::gm::list\_argument, and stan::gm::categorical\_argument.

Definition at line 67 of file argument.hpp.

|  |  |  |  |  |  |
| --- | --- | --- | --- | --- | --- |
| int stan::gm::argument::compute\_indent | ( | const int | *depth* | ) |  |

inline

Definition at line 71 of file argument.hpp.

|  |  |  |  |  |
| --- | --- | --- | --- | --- |
| std::string stan::gm::argument::description | ( |  | ) | const |

inline

Definition at line 30 of file argument.hpp.

|  |  |  |  |
| --- | --- | --- | --- |
| virtual void stan::gm::argument::find\_arg | ( | std::string | *name*, |
|  |  | std::string | *prefix*, |
|  |  | std::vector< std::string > & | *valid\_paths* |
|  | ) |  |  |

inlinevirtual

Reimplemented in stan::gm::categorical\_argument, stan::gm::list\_argument, and stan::gm::singleton\_argument< T >.

Definition at line 46 of file argument.hpp.

|  |  |  |  |  |
| --- | --- | --- | --- | --- |
| std::string stan::gm::argument::name | ( |  | ) | const |

inline

Definition at line 26 of file argument.hpp.

|  |  |  |  |
| --- | --- | --- | --- |
| virtual bool stan::gm::argument::parse\_args | ( | std::vector< std::string > & | *args*, |
|  |  | std::ostream \* | *out*, |
|  |  | std::ostream \* | *err*, |
|  |  | bool & | *help\_flag* |
|  | ) |  |  |

inlinevirtual

Reimplemented in stan::gm::categorical\_argument, stan::gm::singleton\_argument< T >, stan::gm::list\_argument, and stan::gm::unvalued\_argument.

Definition at line 37 of file argument.hpp.

|  |  |  |  |
| --- | --- | --- | --- |
| virtual void stan::gm::argument::print | ( | std::ostream \* | *s*, |
|  |  | const int | *depth*, |
|  |  | const std::string | *prefix* |
|  | ) |  |  |

pure virtual

Implemented in stan::gm::list\_argument, stan::gm::categorical\_argument, stan::gm::unvalued\_argument, and stan::gm::valued\_argument.

|  |  |  |  |
| --- | --- | --- | --- |
| virtual void stan::gm::argument::print\_help | ( | std::ostream \* | *s*, |
|  |  | const int | *depth*, |
|  |  | const bool | *recurse* |
|  | ) |  |  |

pure virtual

Implemented in stan::gm::list\_argument, stan::gm::categorical\_argument, stan::gm::valued\_argument, and stan::gm::unvalued\_argument.

|  |  |  |  |
| --- | --- | --- | --- |
| virtual void stan::gm::argument::probe\_args | ( | argument \* | *base\_arg*, |
|  |  | std::stringstream & | *s* |
|  | ) |  |  |

inlinevirtual

Reimplemented in stan::gm::categorical\_argument, stan::gm::list\_argument, and stan::gm::singleton\_argument< T >.

Definition at line 44 of file argument.hpp.

|  |  |  |  |
| --- | --- | --- | --- |
| static void stan::gm::argument::split\_arg | ( | const std::string & | *arg*, |
|  |  | std::string & | *name*, |
|  |  | std::string & | *value* |
|  | ) |  |  |

inlinestatic

Definition at line 54 of file argument.hpp.

## Member Data Documentation

std::string stan::gm::argument::\_description

protected

Definition at line 77 of file argument.hpp.

std::string stan::gm::argument::\_name

protected

Definition at line 76 of file argument.hpp.

int stan::gm::argument::help\_width

protected

Definition at line 80 of file argument.hpp.

int stan::gm::argument::indent\_width

protected

Definition at line 79 of file argument.hpp.

---

The documentation for this class was generated from the following file:

- src/stan/gm/arguments/argument.hpp


---

[ Stan Home Page ]

*© 2011–2014,
Stan Development Team.*
